# Vitamin D and vitamin D binding protein levels in COVID-19 intensive care unit patients: A prospective multicenter study

**DOI:** 10.5937/jomb0-47822

**Published:** 2024-06-15

**Authors:** Vesile Örnek Diker, Gülseren Yılmaz, Muhammed Emin Düz, Mürvet Algemi, Mehmet Köseoğlu, Hümeyra Öztürk Emre, Osman Oğuz

**Affiliations:** 1 Mehmet Akif Ersoy T.C.S. Training and Research Hospital, Medical Biochemistry, Istanbul, Turkey; 2 Kanuni Sultan Süleyman Training and Research Hospital, Anesthesia and Reanimation, Istanbul, Turkey; 3 Amasya University, Sabuncuoglu Şerefeddin Training and Research Hospital, Medical Biochemistry, Amasya, Turkey; 4 Kanuni Sultan Süleyman Training and Research Hospital, Medical Biochemistry, Istanbul, Turkey; 5 Mehmet Akif Ersoy T.C.S. Training and Research Hospital, Anesthesia and Reanimation, Istanbul, Turkey; 6 Basaksehir Çam and Sakura City Hospital, Medical Biochemistry, Izmit, Turkey; 7 American Hospital, Clinical Biochemistry, Istanbul, Turkey

**Keywords:** COVID-19, vitamin D, vitamin D binding protein, intensive care unit, SARS-CoV-2, COVID-19, vitamin D, vezujući protein za vitamin D, jedinica intenzivne nege, SARS-CoV-2

## Abstract

**Background:**

Vitamin D binding protein plays a crucial role in regulating vitamin D levels by carrying vitamin D and its metabolites and immunological response by binding to endotoxins and fatty acids. We aimed to compare vitamin D, DBP, and specific inflammatory markers among intensive care unit (ICU) patients with and without the COVID19 virus.

**Methods:**

This multicenter study in two training and research hospitals included 37 (13 female) COVID-19positive and 51 (34 female) COVID-19-negative ICU patients. 25(OH) vitamin D, DBP, C-reactive protein (CRP), procalcitonin (PCT), D-dimer, troponin T (TnT), interleukin 6 (IL-6) and ferritin levels, survival, mortality rates, duration of stay (ICU) were examined.

**Results:**

We observed higher ferritin and CRP levels, along with lower DBP, TnT, and D-dimer levels, in patients with COVID-19. ICU patients with COVID-19 exhibited elevated mortality rates (Odds Ratio: 3.012, 95% Confidence Interval (1.252-7.248), p=0.013). However, no statistically significant correlation was observed between mortality rates and Vitamin D or DBP levels across the ICU patient cohort.

**Conclusions:**

Vitamin D values were found to be low in all intensive care patients, regardless of their COVID-19 status. Contrary to the literature, COVID-19 patients had lower D-dimer and TNT levels than negative controls. However, COVID-19-positive ICU patients have decreased DBP. Further, DBP gene polymorphism studies are needed to explain this situation.

## Abstract (sr)

**Uvod:**

Protein koji vezuje vitamin D igra ključnu ulogu u regulisanju nivoa vitamina D tako što nosi vitamin D i njegove metabolite i imunološki odgovor vezivanjem za endotoksine i masne kiseline. Cilj nam je bio da uporedimo vitamin D, DBP i specifične inflamatorne markere među pacijentima na jedinici intenzivne nege (ICU) sa i bez virusa COVID-19.

**Metode:**

Ova multicentrična studija u dve bolnice za obuku i istraživanje uključivala je 37 (13 žena) pozitivnih na COVID19 i 51 (34 žene) pacijenata na intenzivnoj intenzivnoj nezi sa COVID-19 negativnim. 25(OH) vitamin D, DBP, C-reaktivni protein (CRP), prokalcitonin (PCT), D-dimer, troponin T (TnT), nivoi interleukina 6 (IL-6) i feritina, preživljavanje, stope mortaliteta, trajanje boravak (ICU) su pregledani.

**Rezultati:**

Primetili smo više nivoe feritina i CRP, zajedno sa nižim nivoima DBP, TnT i D-dimera, kod pacijenata sa COVID-19. Pacijenti sa intenzivne nege sa COVID-19 su pokazali povišene stope mortaliteta (odnos šanse: 3,012, 95% interval poverenja [1,252-7,248], p=0,013). Međutim, nije primenjena statistički značajna korelacija između stopa mortaliteta i nivoa vitamina D ili DBP u kohorti pacijenata na intenzivnoj nezi.

**Zaključak:**

Utvrđeno je da su vrednosti vitamina D niske kod svih pacijenata na intenzivnoj nezi, bez obzira na njihov COVID-19 status. Suprotno literaturi, pacijenti sa COVID-19 imali su niže nivoe D-dimera i TNT-a od negativnih kontrola. Međutim, pacijenti sa intenzivne nege pozitivni na COVID19 imaju smanjen DBP. Dalje, potrebne su studije polimorfizma DBP gena da bi se objasnila ova situacija.

## Introduction

Vitamin D, a cholesterol-derived steroid hormone, is vital for musculoskeletal health and immune function [1]. Vitamin D also affects respiratory viral infections [2]. Vitamin D deficiency also worsens respiratory issues, such as bronchiolitis, in neonates requiring special care [3]. Vitamin D deficiency also potentiates proinflammatory cytokines, including Tumor necrosis factor (TNF), type 1 interferons (IFN), and IL-6 [4] [5]. One of the probable variables influencing COVID-19 risks and outcomes has been identified as insufficient vitamin D levels [6]. Recently, researchers explored the impact of vitamin D insufficiency and the significance of vitamin D supplementation in COVID-19 patients [7]. Vitamin D may prevent viral entry by interacting with the angio ten sinconverting enzyme-2 receptor (ACE2), the virus’s entry site, owing to its (S) protein spike [8]. Vitamin D also controls macrophages to prevent cytokine storms [9]. Vitamin D supplementation has shown to have no significant influence on the probability of COVID-19 infection, although it did exhibit protective benefits against mortality and ICU admission in COVID-19 patients, according to a systematic review and meta-analysis [10].

Vitamin D binding protein (DBP) binds 85–90% of circulating vitamin D molecules under normal physiological circumstances [11]. DBP also activates macrophages, enhances the leukocyte chemotactic potential of activated complement peptides, scavenges actin, and transports fatty acids [12]. After hyperinflammation and complement system activation, COVID-19 pneumonia patients may develop acute respiratory distress syndrome (ARDS), which involves the C5a-C5aR axis. DBP has been discovered in ARDS patients’ bronchoalveolar lavage fluid. DBP release boosts monocyte and neutrophil recruitment, aggregation, and activation at endothelium damage sites, causing an oxidative burst. 25(OH) vitamin D and 1,25(OH)2 vitamin D may impede this chemotaxis by competing for DBP’s binding site, influencing the illness’s course and prognosis [13]. Extracellular globular actin (G-actin) polymerizes into filamentous actin (F-actin) after COVID-19 cell death and lung tissue injury. In ARDS patients’ sera, F-actin is linked to microembolisms, pulmonary vascular angiopathy, and multiple organ failure syndrome. Under stress, SARS-CoV can induce apoptosis and actin rearrangement in mammalian cells [14]. DBP and gelsolin cleave actin and impede its repolymerization as components of the extracellular actin scavenger system. However, excessive concentrations and/or extended exposure to VDBP-actin complexes may trigger endothelial cell damage and death, notably in the microvasculature of the lungs [15].

## Materials and methods

### Patients

This multicenter study was conducted at two separate training and research hospitals with 37 (13 females) COVID-19-positive and 51 (34 females) negative patients hospitalized in the ICU between July 2021 and April 2022. COVID-19 status was confirmed by the polymerase chain reaction (PCR) method for the negative and positive groups. Patients were hospitalized in separate ICU rooms (Covid ICU and non-Covid ICU). The following ICU criteria were established for COVID-19-positive patients: respiratory rates ≥ 30 breaths per minute, oxygen saturation 93%, lung infiltrates > 50%, and at high risk for clinical deterioration and for developing a critical illness, including acute respiratory distress syndrome (ARDS) [16]. For COVID-19-negative patients, an adapted guideline from the London Department of Health was used for ICU criteria [17]. Blood samples drawn from the patients within three days of ICU hospitalization were taken into gel tubes without additives and EDTA-K3 tubes. Then, the tubes were centrifuged at 1500*g for 15 minutes to obtain serum and plasma. Afterward, serum and plasma samples were kept at -80 degrees Celsius until the study day. Informed consent was obtained from the patients for the study. Relatives of those unable to give consent signed an informed consent form. The study was carried out following the Declaration of Helsinki. Ethics committee approval was obtained on 08/11/2022 with the number 2022-63.

### Study design

COVID-19-positive and negative ICU patients hospitalized in two training and research hospitals were divided into two groups. Survival, ICU length of stay, 25(OH) vitamin D, vitamin D binding protein (DBP), CRP, procalcitonin (PCT), D-dimer, troponin-t (TnT), interleukin-6 (IL-6), and ferritin levels were analyzed. These markers were examined in terms of mortality and COVID-19. In addition, comparative analyses based on age and gender were performed. The working methods of the relevant parameters and the devices used are presented in Table 1. The study was started after the patients were admitted to the ICU, and the data were created prospectively, not from a retrospective file but from the data obtained as a result of patient follow-up.

**Table 1:** The parameters studied and the devices used are indicated in the list. Vit-D, 25-OH vitamin D; DBP, Vitamin D binding protein; PCT, Procalcitonin; TnT, Troponin-t; IL-6, Interleukin-6, ECLIA, Electrochemiluminescence Immunoassay; FEU, Fibrinogen Equivalent Unit.

Test	Method	Range	Unit	Device	Reactive
Ferritin	ECLIA	Female: 13–150	μg/L	Roche cobas c 702 and<br>cobas e 801	Roche Diagnostics
		Male: 30–400			Sandhofer Strasse,
IL-6	ECLIA	0-7	pg/mL	Roche cobas c 702 and<br>cobas e 801	Roche Diagnostics
					Sandhofer Strasse,
TnT	ECLIA	<14	ng/L	Roche cobas c 702 and<br>cobas e 801	Roche Diagnostics
					Sandhofer Strasse,
CRP	ECLIA	<5	mg/L	Roche cobas c 702 and<br>cobas e 801	Roche Diagnostics
					Sandhofer Strasse,
D-dimer	İmmüno<br>türbidimetrik	0–0.50	μg/mL FEU	Sysmex cs2500	Innovance, Siemens
					Erlangen, Germany
PCT	ECLIA	<0.5	μg/L	Roche cobas c 702 and<br>cobas e 801	Roche Diagnostics
					Sandhofer Strasse,
Vit-D	ECLIA	20-80	μg/L	Roche cobas c 702 and<br>cobas e 801	Roche Diagnostics
					Sandhofer Strasse,
DBP	ECLIA	104-477	μg/mL	Thermo scientific Multiskan	SunRed, Baoshan
				Massachusetts, USA	Shanghai, China

### Statistics

Frequency and percentage values were given for categorical variables. Mean, standard deviation, median, minimum, and maximum values were given for continuous variables. The normal distribution of variables was tested with the Kolmogorov-Smirnov test. The Wilcoxon test was used to compare non-parametric group means because no two samples are produced from the same population. The paired sample t-test was employed to compare parametric group means for two different categories since the variances of the two groups are assumed to be equal. The correlation between parameters was analyzed by the Spearman test. An odds ratio (OR) is calculated with logistic regression analysis for the association between COVID-19 and mortality. ROC curves were used to examine the effects of vitamin D and DBP levels on mortality. A p-value < 0.05 was considered significant. Statistical analyses were assessed via SPSS 26 software (IBM Inc, Illinois, USA).

## Results

In the COVID-19-positive group, 35% of the participants were female, compared to 66% in the opposing group. There were no age differences between groups (61.3±16.6 vs. 64.4±19.7, p=0.439). While DBP, D-dimer, and TNT values were lower in COVID-19 patients compared to the other group, CRP and ferritin values were higher. ICU stay was shorter in COVID-19 patients. Descriptive statistics are presented in Table 2. Both ICU groups had inadequate vitamin D levels (<20 ng/L). According to the chi-square test, OR: 3.012, with 95%CI (1.252–7.248), was detected in terms of the effect of COVID-19 on mortality in ICU patients (p=0.013). In Pearson correlation analysis, a significant positive correlation was found between DBP and ICU stay (r=0.628, p<0.001). However, no meaningful relationship existed between Vitamin D and DBP levels and mortality in all ICU patients or the COVID-19-positive group. Table 3 shows the relationship between Vitamin D and DBP and mortality in all ICU patients, and Table 4 in COVID-19-positive ICU patients. Neither parameter provided satisfactory AUC values. The linear regression analysis within COVID-19 positive and negative groups found no correlation between vitamin D and the inflammatory parameters CRP, D-dimer, and TNT (p=0.552, p=0.428, and p=0.466, respectively). Similarly, no association was found between DBP and CRP, D-dimer, and TNT (p=0.247, p=0.176, and p=0.066, respectively).

**Table 2:** Descriptive statistics. Vit-D, 25-OH vitamin D; DBP, Vitamin D binding protein; PCT, Procalcitonin; TnT, Troponin-t; IL-6, Interleukin-6; 0, COVID- 19 negative; 1, COVID-19 positive.

	COVID-19	Mean	Std. Deviation	Minimum	Maximum	p-value
Age	0	64.431	19.791	18.000	92.000	0.439
	1	61.324	16.600	30.000	92.000	
Vitamin D	0	10.828	8.184	3.000	42.300	0.128
	1	14.780	15.688	3.000	89.800	
DBP	0	603.892	286.758	115.070	1.956.420	0.007
	1	448.197	220.709	170.140	1.418.380	
CRP	0	98.687	88.914	1.260	318.020	0.008
	1	155.382	106.442	22.330	406.230	
PCT	0	9.981	6.611	1.840	24.250	0.745
	1	20.836	51.488	1.590	322.100	
D-dimer	0	6.292	7.503	0.300	35.200	0.013
	1	2.688	4.698	0.390	27.000	
TNT	0	87.744	180.429	3.600	1.224.000	0.036
	1	23.289	38.649	1.300	223.000	
IL-6	0	99.206	88.887	4.420	334.300	0.294
	1	376.083	1.380.883	4.240	8.336.000	
Ferritin	0	494.705	516.234	11.900	2.078.000	0.016
	1	1.096.116	1.450.304	61.600	7.753.000	
Days of ICU stay	0	21.549	19.584	1.000	78.000	0.037
	1	13.892	11.556	3.000	58.000	

**Table 3:** ROC analysis shows the relationship between Vitamin D and DBP and mortality in all ICU patients. a. Under the non-parametric assumption b. Null hypothesis: true area = 0.5

Test Result Variable(s)	Area	Std. Error^a^	Asymptotic Sig.^b^	Asymptotic 95% Confidence Interval	
				Lower Bound	Upper Bound
Vitamin D	0.545	0.062	0.472	0.422	0.667
DBP	0.555	0.063	0.373	0.432	0.678

**Table 4:** ROC analysis demonstrates the relationship between Vitamin D and DBP and mortality in COVID-19-positive ICU patients. a. Under the non-parametric assumption b. Null hypothesis: true area = 0.5

Test Result	Area	Std. Error^a^	Asymptotic Sig.^b^	Asymptotic 95% Confidence Interval	
				Lower Bound	Upper Bound
Vitamin D	0.575	0.097	0.452	0.385	0.764
DBP	0.453	0.101	0.639	0.254	0.652

## Discussion

In the present study, we investigated the association of vitamin D and DBP levels with ICU stays and mortality rates in COVID-19-positive and negative patients. The absence of a difference in vitamin D levels between ICU groups was the first of our most noteworthy study findings. A comprehensive review and meta-analysis of 31 observational studies suggested that a correlation between low blood 25(OH) vitamin D levels and COVID-19-related health outcomes may be interpreted as a trend but was not statistically significant [18]. Although these meta-analysis results were consistent with our study, the literature had counterexamples. Patients with low vitamin D levels had an increased risk of ARDS necessitating ICU admission or fatality owing to SARS-CoV-2 infection, increased susceptibility to SARS-CoV-2 infection, and accompanying hospitalization, according to a meta-analysis of 54 studies [19]. In addition, vitamin D insufficiency is related to a more severe COVID-19 infection, according to a second meta-analysis of 17 trials, including 2,756 individuals [20]. Besides, in another systematic review of 21 studies, researchers found that low serum vitamin D levels increase the likelihood of contracting COVID-19 but no change in mortality [21]. Moreover, both groups had insufficient vitamin D levels and showed no difference in mortality. The research we mentioned emphasized clinical deterioration in COVID-19 patients and was worried about low levels of vitamin D. ICU patients who tested positive and negative for COVID-19 were not compared. It is recognized that there was no correlation between COVID-19 status and vitamin D levels in ICU patients, yet both groups have low vitamin D levels. Considering the negative consequences of vitamin D deficiency on the immune system, the need for intensive care of patients may be related to low vitamin D levels, even if COVID-19 is negative. Alternatively, it may be thought that vitamin D supplementation is needed in ICU patients, whether due to COVID-19 or not.

We detected lower DBP levels in COVID-19-positive ICU patients. However, researchers say high concentrations and/or extended exposure to DBP-actin complexes may cause endothelial cell damage and death, particularly in the pulmonary microvasculature [15]. They further claimed that DBP regulates vitamin D-induced T cell responses by sequestering 25(OH) vitamin D and blocking 1,25(OH) vitamin D synthesis in T cells [22]. There are several reasons for the inconsistency of our results with the literature. First, to date, DBP is known as the most polymorphic protein, with various alleles significantly influencing its biological activities [23]. Two common DBP alleles, rs7041 and rs4588, have been implicated in the development of various clinical disorders, primarily due to their affinity for vitamin D [23]. On the other side, the rs7041 locus was discovered to be related to increased vulnerability to hepatitis C virus infection [24]. Because DBP gene polymorphisms have been linked to increased vulnerability to infections and vitamin D insufficiency in other populations, they may also have a role in COVID-19 [25]. According to a Turkish study, vitamin D levels were governed mainly by the genetic background of both healthy people and COVID-19 patients. Genetic variants in the DBP gene, notably SNP in the rs7041 locus, were shown to be linked with the frequency of COVID-19 and death rates across nations in their study [26]. Based on this information, we believe the contradiction of the DBP results in our study data with the literature is due to our study group’s DBP gene polymorphisms. Our findings may not be consistent with the literature because various loci may have distinct impacts on DBP levels and interaction with the immune system. Furthermore, because DBP levels impact vitamin D levels, the difference in vitamin D levels across groups may be connected to DBP gene polymorphisms.

Another intriguing finding was that COVID-19 patients had lower D-dimer and TNT levels than the other group. A meta-analysis of 29 studies (4,328 patients) found that high D-dimer levels upon admission are linked to greater illness severity and the likelihood of mortality in SARS-CoV-2 patients [27]. Furthermore, another meta-analysis indicated that SARS-CoV-2 infected patients with increased D-dimers tend to have poorer clinical outcomes (all-cause death, ICU hospitalization, or ARDS), so D-dimer measurement might assist clinical decision-making [28]. In particular, researchers recommended investigations revealing elevated TNT levels in COVID-19 patients [29]. The fact that the D-dimer and TNT values of the COVID-19 patients in our study were lower than the other group may be a marker of a more severe systemic inflammatory process in COVID-19-negative patients. In addition, it should be remembered that these parameters increased slightly in the COVID-19 group compared to the other group. In addition, vitamin D and DBP levels did not correlate with inflammatory parameters, according to our study data. However, vitamin D levels were insufficient in both groups. At this point, the importance of vitamin D supplementation comes to mind. Interestingly, however, as a result of a systematic review and meta-analysis, researchers found that the rates of RT-PCR positivity were significantly decreased in the vitamin D intervention group compared to the non-vitamin D groups [30]. In conclusion, patients with COVID-19 who take vitamin D supplements had lower rates of intensive care unit (ICU) admission, death, and RT-PCR positive [30]. In light of the literature data, we interpret that intense inflammatory processes developing in ICU patients may have different effects on D-dimer and TNT, with or without COVID-19. On the other hand, according to our study results, ICU deaths caused by COVID-19 are three times higher than other ICU patients. At this point, it can be thought that severe hypoxia and lung involvement, which may develop in the ICU, are more common in COVID-19 patients.

### Limitations

Our failure to classify the reason for non-COVID-19 ICU admissions was the primary omission. The inability to regularly investigate these markers in patients rendered it hard to track the clinical progress. The small size of our patient groups was another of our limitations.

## Conclusions

Vitamin D levels are low in ICU patients independent of COVID-19 but do not directly affect mortality. We observed that as DBP levels increase, the length of stay in the ICU also increases. Our findings, which contradict the existing literature, show that DBP is significantly lower in COVID-19-positive ICU patients than negative ones. This may be traced back to variations in the DBP gene, called polymorphisms. We also find that D-dimer and TNT levels were lower in COVID-19 patients than in negative controls, which contradicts the literature.

### Conflict of interest statement

All the authors declare that they have no conflict of interest in this work.
